# Draft Genome Sequence of *Lactobacillus jensenii* Strain MD IIE-70(2)

**DOI:** 10.1128/genomeA.01005-13

**Published:** 2013-11-27

**Authors:** Andrey V. Karlyshev, Sindhujan Nadarajah, Vyacheslav M. Abramov

**Affiliations:** School of Life Sciences, Faculty of Science, Engineering and Computing, Kingston University, Kingston upon Thames, United Kingdoma; Institute of Immunological Engineering, Lyubuchany, Moscow Region, Russian Federationb

## Abstract

A draft genome sequence of *Lactobacillus jensenii* strain MD IIE-70(2) was determined using Ion PGM technology. The reads were mapped to a reference strain and assembled using a combination of tools. The genetic features revealed in this study will assist in understanding the probiotic properties of *Lactobacillus* bacteria.

## GENOME ANNOUNCEMENT

*Lactobacillus jensenii* predominates in the microflora of a healthy human vagina and plays an important role in protecting the host from urogenital infections (1). This report describes a draft genome sequence of *L. jensenii* strain MD IIE-70(2), selected for its superior probiotic properties compared to other *Lactobacillus* strains tested (V. M. Abramov, V. G. Melnikov, I. V. Kosarev, and V. C. Khlebnikov, unpublished data).

At the time of this writing (October 2013), there were no complete and six draft *L. jensenii* genome sequences: those of *L. jensenii* JV-V16, *L. jensenii* 115-3-CHN, *L. jensenii* 1153, *L. jensenii* 269-3, *L. jensenii* 27-2-CHN, and *L. jensenii* SJ-7A-US, with estimated genome sizes between 1.6 and 1.75 Mb and a G+C content range of 34.1 to 34.5%.

The mapping of 1,403,599 reads generated by two sequencing runs on the Ion Torrent PGM using a 314 chip onto the available draft genome sequences revealed the highest level of similarity to *L. jensenii* strain 1153. The assembly of reads performed by IonTorrent Assembler and CLC Genomics Workbench *de novo* assembly programs resulted in 765 contigs. Mapping of the contigs onto a genome sequence of *L. jensenii* 1153 produced 37 consensus contigs up to 0.4 Mb in size. *De novo* assembly of unmapped reads (8.65%) resulted in 42 contigs. A combination of the latter with consensus sequences, followed by the exclusion of contigs of <1 kb, splitting of misassembled contigs, and elimination of contigs with low coverage, resulted in a final assembly of 60 contigs (1,137 to 404,149 bases) with 113.82-fold coverage. The total size of the assembly (1,759,880 bases) and G+C content (34.3%) are in perfect agreement with figures for the genomes of other strains of this species.

Data annotation was conducted by using the NCBI GenBank annotation pipeline and RAST server (2). The latter reported 1,754 coding sequences and 60 RNAs. Compared with other genomes of this species, the differences found were mainly attributed to prophage-related genes, transposases, and insertion sequence (IS) elements. Five genes encoding putative adhesins (up to 6,013 amino acids in size) were identified. The observed sequence difference with similar adhesins found in other *L. jensenii* strains may reflect adaptation to particular host systems. The capsular polysaccharide-related cluster contains both conserved and variable genes, suggesting interstrain variation in a capsular polysaccharide structure. In common with other *Lactobacillus* spp., the test strain contains a gene encoding pyruvate oxidase that is responsible for H_2_O_2_ production (3). The presence of the genes responsible for resistance to β-lactam antibiotics and fluoroquinolones may be beneficial when combining probiotic treatment with antibiotic therapy, but there may be a risk of horizontal gene transfer of these determinants to pathogenic bacteria.

Further studies of this strain can provide interesting opportunities to investigate a link between the genetic features and functionalities of these bacteria. Comparative genomics studies of different strains of the same species are important because the “probiotic benefits associated with one species or strain do not necessarily hold true for others” (4). A combination of the recent advancement in genetic engineering techniques and the data reported in this study may also assist in the construction of novel beneficial probiotic strains with improved properties.

### Nucleotide sequence accession numbers.

This whole-genome shotgun project has been deposited at DDBJ/EMBL/GenBank under the accession no. AVCU00000000. The version described in this paper is version AVCU01000000.
